# Path-based quantification of activation and repression in Boolean models using BooLEVARD

**DOI:** 10.1038/s41540-025-00605-y

**Published:** 2025-11-19

**Authors:** Marco Fariñas, Eirini Tsirvouli, John Zobolas, Tero Aittokallio, Åsmund Flobak, Kaisa Lehti

**Affiliations:** 1 Department of Biomedical Laboratory Science, Norwegian University of Science and Technology (NTNU), Trondheim, Norway; 2 Department of Biology, Norwegian University of Science and Technology (NTNU), Trondheim, Norway; 3 Department of Clinical and Molecular Medicine, Norwegian University of Science and Technology (NTNU), Trondheim, Norway; 4 Department of Cancer Genetics, Institute for Cancer Research, Oslo University Hospital (OUH), Oslo, Norway; 5 Oslo Centre for Biostatistics and Epidemiology (OCBE), University of Oslo, Oslo, Norway; 6 Institute for Molecular Medicine Finland (FIMM), HiLIFE, University of Helsinki, Helsinki, Finland; 7 The Cancer Clinic, St. Olavs Hospital, Trondheim, Norway; 8 Department of Biotechnology and Nanomedicine, Sintef Industry, Trondheim, Norway; 9 Department of Microbiology, Tumor and Cell Biology, Karolinska Institute (KI), Stockholm, Sweden; 10 Present Address: NEC OncoImmunity AS, Oslo, Norway

**Keywords:** Systems biology, Computer modelling, Dynamic networks, Dynamical systems, Signal processing, Software

## Abstract

Boolean models are a powerful resource for studying dynamic processes of biological systems. However, their inherent discrete nature limits their ability to capture continuous aspects of signal transduction, such as signal strength or protein activation levels. Although existing tools provide some path exploration capabilities that can be used to explore signal transduction circuits, the computational workload often requires simplifying assumptions that compromise the accuracy of the analysis. Here, we introduce BooLEVARD, a Python package designed to efficiently quantify the number of paths leading either to node activation or repression in Boolean models, which offers a more detailed and quantitative perspective on how molecular signals propagate through signaling networks. By focusing on the collection of non-redundant paths directly influencing Boolean outcomes, BooLEVARD enhances the precision of signal strength representation. We showcase the application of BooLEVARD in studying cell-fate decisions using a Boolean model of cancer metastasis, demonstrating its ability to identify critical signaling events. In addition, through a second use case, we demonstrated BooLEVARD’s capability to scale efficiently across increasingly large and connected Boolean models. Through these properties, BooLEVARD provides a distinctive tool for quantitative analysis of signaling dynamics within Boolean models, which can increase our understanding of disease development and drug responses. BooLEVARD is freely available at https://github.com/farinasm/boolevard.

## Introduction

The rapid growth of high-throughput technologies has enabled the generation of vast amounts of biological data, leading to a critical challenge of its analysis and interpretation^1^. Computational modeling methods have provided enhanced understanding of biological processes, supporting experimental research. There are several types of modeling formalisms, each describing biological systems in a varying level of detail and requiring different types of information and data^2^. Examples of such mathematical formalisms include Boolean equations, constraint-based modeling (CBM), or differential equations^3–6^. While differential equations are particularly useful for modeling continuous processes, such as biochemical reactions, molecular transport, or diffusion, their use is often limited by the requirement for detailed kinetic experimental data, which are typically hard to acquire^6^. Boolean equations and CBMs are more easily applicable to discrete processes. Even though CBMs, like genome-scale metabolic modeling, also require certain parameters, such as omics data and culture media composition, these are easier to obtain^3^. Boolean models are typically based on signaling diagrams representing events such as transcriptional regulation and protein-protein interactions. Boolean models do not require any kinetic data and can be constructed based on causal interactions^4^. As such, Boolean modeling provides a simplified representation of dynamic systems, while still effectively capturing the key mechanisms that govern biological processes and lead to cellular phenotypes^7^. Moreover, their less stringent information requirements make them a practical choice for modeling a wide range of biological systems.

Boolean models consist of a network of interacting nodes, each representing a biological entity that can exist in one of two states: active (i.e., 1) or inactive (i.e., 0)^4^. The state of a node, referred to as its Boolean or local state, is governed by Boolean equations that define how the states of upstream regulators are integrated, and depend on the network’s topology^8^. During the model simulation, the user can optionally specify an initial condition (i.e., a set of active and inactive nodes), from which the model can explore all possible state transitions. Single states or subsets of states that have no outgoing transitions are known as attractors, which can be further classified into stable states or cyclic attractors, respectively, and are expressed as Boolean vectors of local states (i.e., a series of stably active and inactive components). Stable states have been shown to be frequently associated with cellular phenotypes^9^. Attractor reachability, starting from given initial conditions until the attractors are reached, can be explored with several computational tools available for analyzing Boolean models. Examples include bioLQM, mpbn, Pint, and GINsim^10–14^. Other tools, such as MaBoSS and PhysiBoSS^15–17^, extend Boolean modeling by incorporating continuous Markov chains and allow the study of a system’s evolution over pseudo-time, and explore the probabilities of node activities and stable state reachability^15^. Furthermore, concepts such as logic hypergraph^18^, Elementary Signaling Modes (ESMs)^19^, or Logic Backbone^20^ focus on scratching structural or minimal pathway motifs, providing insights on how signals are transduced within the models.

Despite their advantages, Boolean models inherently provide only binary information (whether a node is active or inactive), without capturing the intensity of activation or inhibition^21^. To address this limitation, we introduce BooLEVARD (Boolean Logical Evaluation of Activation and Repression in Directed pathways), a Python package that leverages equations within Boolean models to systematically count the number of activating and repressing paths leading to the local state of a given node. Unlike existing approaches, which typically do not quantify repression and often require users to impose a maximum path length constraint to manage computational complexity^12,22^, BooLEVARD enables the exploration of activating and inhibitory signal intensities. In the present study, we demonstrate how signal transduction strength influences invasive and apoptotic fate decisions using a Boolean model of cancer metastasis^23^. Moreover, perturbation performance and downstream analysis with BooLEVARD allowed for a more detailed and granular distribution of the perturbation impact. To ensure that these insights are attainable at scale, we conducted a comprehensive scalability analysis across a collection of synthetic Boolean models, showing that BooLEVARD maintains practical runtimes and path enumeration even in large, highly connected models. Overall, this approach provides a quantitative extension to traditional stable-state analysis, thereby broadening the scope of Boolean modeling for dissecting the complexity of biological systems.

## Results

To evaluate the capabilities of BooLEVARD, we present three complementary analyses in this section. Together, these use cases demonstrate both the biological insight and the practical performance advantages offered by BooLEVARD.

### BooLEVARD reveals variability in signal transduction strength, influencing cell-fate decisions

To first investigate how differences in signal transduction strength can be revealed across stable states that share identical phenotypic outputs, and to test the importance of considering the signal strength in modeling biological processes, we applied BooLEVARD to a Boolean model of cancer metastasis^23^ (Supplementary Code 1). This Boolean model, developed by Cohen et al.^23^, incorporates key signaling cascades driving cell invasion and apoptosis in cancer, focusing on ECM- and DNA-damage-induced cell functions. Accordingly, it includes two input nodes (*DNAdamage*, *ECMicroenv*) and six phenotype nodes (*Invasion*, *Migration*, *CellCycleArrest*, *Apoptosis*, *Metastasis*, and *EMT*). The Metastasis node was not included in the analysis, as its activation is exclusively dependent on the Migration node. In addition, the *TGFbeta* node was split into two nodes, namely *TGFbeta_i* (activated by *NICD* and inhibited by *CTNNB1*) and *TGFbeta_e* (activated by *ECMicroenv*), to differentiate between autocrine- and paracrine-TGFβ-related regulation of the cancer cell signaling, respectively. Both *TGFbeta_i* and *TGFbeta_e* converge into a generic *TGFbeta* node.

**Fig. 1:**
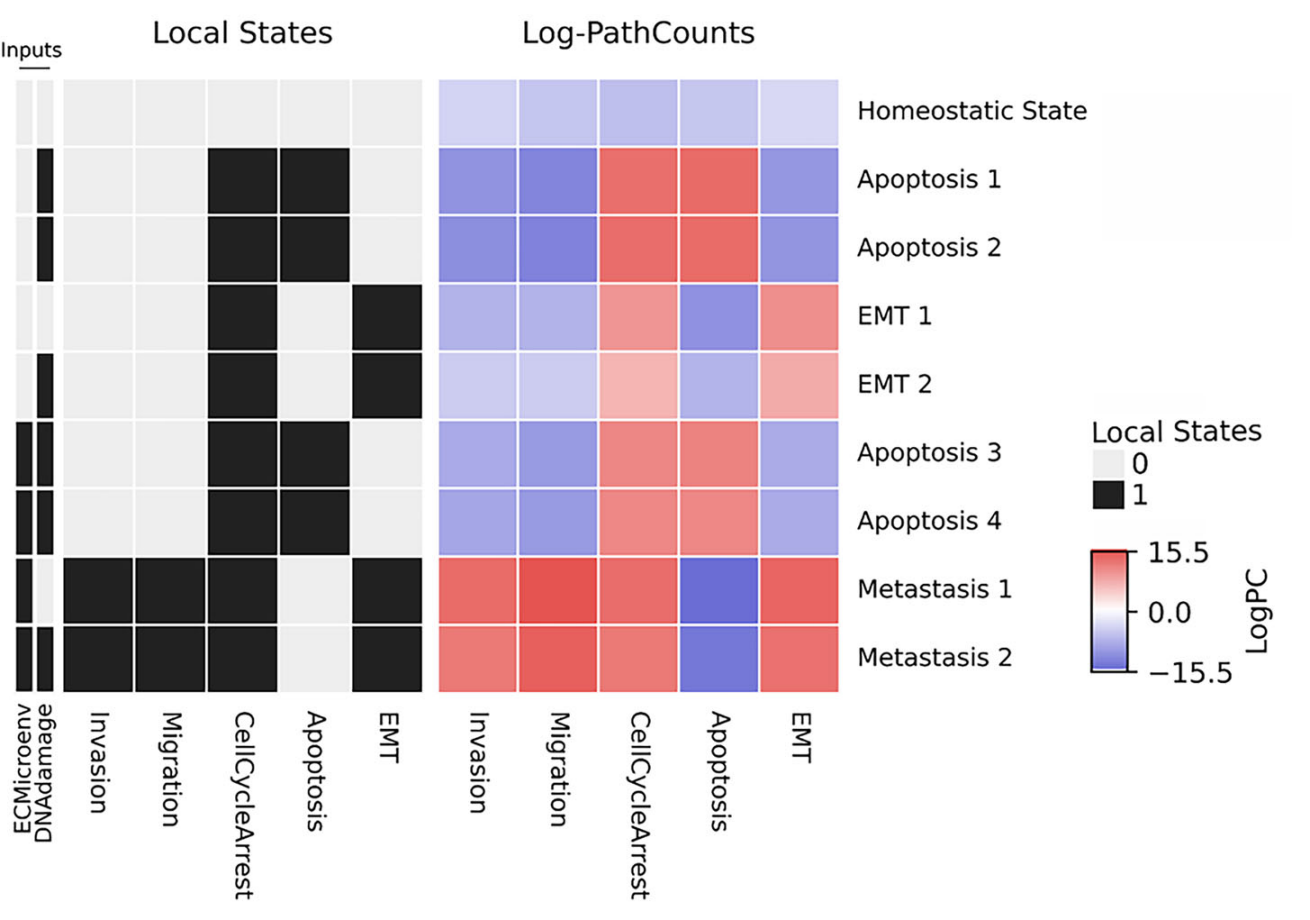
Heatmap showing the Boolean states (left) and the corresponding path counts (right), calculated using BooLEVARD, leading these states for the phenotype nodes in the stable states reached by the metastatic model. The Boolean states of the *DNAdamage* and *ECMicroenv* input nodes driving each stable state are also displayed. Path counts are presented on a logarithmic scale. Active and inactive nodes are presented in black and white, respectively. Active paths triggering node activation and inactivation are presented in red and blue, respectively.

The model produced nine stable states, including one homeostatic state (*HS*, all phenotype nodes are inactive), four apoptotic nodes (*Apop1-4*, *Apoptosis* is active), two EMT states (*EMT1/2*, *EMT* is active), and two metastatic states (*M1/2*, *Invasion* and *Migration* are active). Although stable states within the same phenotypic category shared overlapping local states of phenotype nodes, analysis using BooLEVARD provided greater resolution (Fig. 1). To extend these observations, we developed a script to systematically perform this analysis in Boolean models from the Cell Collective repository^24^ (Supplementary Code 2). A clear example relied on the apoptotic stable states, which could be classified into two groups, integrated by *Apop1/2* and *Apop3/4*, respectively. *Apop1/2* were triggered upon the presence of *DNAdamage* and the absence of *ECMicroenv*, whereas *Apop3/4* were reached upon the presence of both input nodes. Although all four states displayed identical cell fates, as indicated by the Boolean state of the phenotype nodes, BooLEVARD provided additional insight. By counting activatory and inhibitory paths, it predicted reduced apoptotic signaling upon the presence of *ECMicroenv*. The same applied when comparing the *EMT1/2* states, where stronger EMT commitment was observed upon communication with the extracellular microenvironment. These results align well with published experimental findings showing that cancer-ECM crosstalk confers protection against apoptosis^25^. Moreover, cancer cells can undergo EMT in response to DNA damage, thus displaying increased DNA-repair potential. On the other hand, accumulation of genomic damage has been shown to have the opposite effect and trigger apoptosis^26,27^. These results suggest that BooLEVARD’s path-counting approach can reveal differential activating and inhibitory signal transduction strengths in a biologically relevant manner across stable states that share identical phenotypic outputs.

### BooLEVARD highlights the negative correlation between invasion and apoptosis

To investigate whether perturbations with seemingly similar stable states, and therefore phenotypic outcomes, differ in their underlying signaling circuits, we individually perturbed each non-input and non-phenotype node in the model by simulating both additive activation and inhibition scenarios (see *Model Perturbations*). In each case, we analyzed only those stable states in which the perturbation node (i.e., the added activator or inhibitor) was effectively active (set to 1). This ensured that the observed effect on the target node could be attributed to the intended perturbation (always active upon activatory perturbations and always inactive upon inhibitory perturbations). Accordingly, this approach simulates the effects of drug or other types of treatments, either activating or inhibiting the proteins represented in the model. Using BooLEVARD, we evaluated the impact of these perturbations on metastasis by focusing on the Invasion and Apoptosis phenotype nodes as readouts. A total of 195 stable states were analyzed across 48 perturbations, all performed on a local computer with 16 GB RAM and an AMD Ryzen 75800H processor in approximately 21 min (Supplementary Fig. 1).

**Fig. 2:**
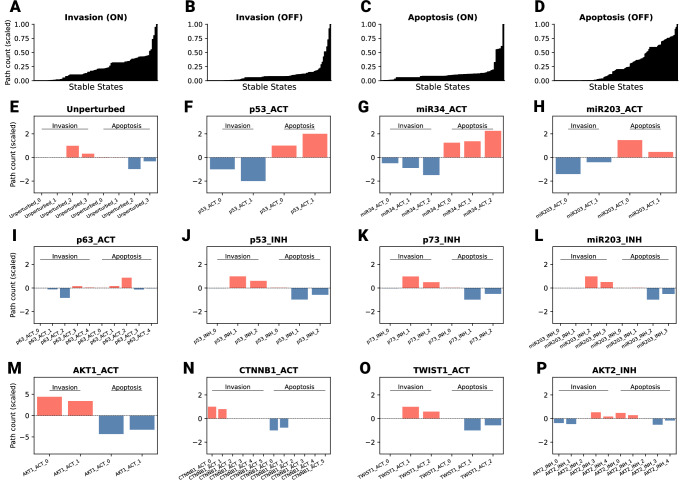
Path-based characterization of invasion and apoptosis phenotypes across stable states and perturbations. **A**–**D** Path count range leading to the Boolean states of the *Invasion* and *Apoptosis* phenotype nodes across the stable states reached under the different perturbations. Path counts are shown as min-max scaled values across all stable states. **E**–**P** Barplots of path counts triggering the activation and inactivation of *Invasion* and *Apoptosis* phenotype nodes across the stable states reached upon perturbation. Each bar shows the min-maxed scaled (with reference at zero) path counts in a given stable state. Activatory (red) and inhibitory (blue) paths are displayed for each target node (*Invasion* and *Apoptosis*) in different perturbations.

Results were then compared to the Boolean states of *Invasion* and *Apoptosis* nodes to identify any differences both stable-state-wise and model-wise (i.e., averaging node Boolean states and path counts of the stable states of a given model). The metastatic states, *M1* and *M2*, were only reached when the *ECMicroenv* input node was activated, which also triggered two apoptotic states. Consequently, the effect of these perturbations on metastatic fate was analyzed under this initial condition. As expected, implementation of BooLEVARD resulted in high granularity across the path count distribution displayed by the perturbations (Fig. 2A–D, Supplementary Figs. 2–4). BooLEVARD allowed for tracing the intensities through which signals were transduced towards the Boolean state of a given node. As a result, differences emerged although two nodes shared the same Boolean state upon different perturbations, which directly highlighted differences between the internal circuits and signal transduction strengths. For example, while perturbations resulting in the highest fate transitions modulating invasion and apoptosis (Fig. 2E–P, Supplementary Fig. 5) showed high agreement between the conventional analysis of node Boolean states and BooLEVARD’s path-counting approach, BooLEVARD resulted in distinctions in the observed intensity of these effects.

Notably, the stable state analysis highlighted *p53_ACT*, *miR34_ACT*, and *miR203_ACT* (Fig. 2F–H) as, on average, the most impactful perturbations in dampening invasion and triggering apoptosis, all three with qualitatively identical effects (Supplementary Fig. 2). Together with *p63_ACT* (Fig. 2I), these perturbations were the strongest triggering anti-invasive fate when using BooLEVARD. Therefore, despite overlapping invasive and apoptotic fate trends, an increasing gradient was observed in *miR203_ACT*, *p53_ACT*, and *miR34_ACT*, respectively. Consistent with the lowest values observed for apoptosis resistance and invasion, miR-34 has been demonstrated to enhance p53-mediated responses and to inhibit proliferation and invasion in vivo^28^. p63, a gene related to p53, is often dysregulated in cancer and overexpression of its transactivation-domain-containing isoform (TAp63) has been linked to enhanced apoptosis and reduced metastatic potential^29^ and, together with p73, it cooperates with p53 to trigger its function^30^. Conversely, stable states reached upon inhibitions of p53, p73, and miR203 cell cycle regulators, and as supported by experimental evidence^31^, the activation of AKT1 and EMT-related genes such as CTNNB1 and TWIST1 (Fig. 2J–O) were predicted to have the strongest impact on promoting invasion and inhibiting apoptosis.

Although not frequent, some mismatches between the approaches with and without BooLEVARD were observed. An interesting case is the simulated inhibition of AKT (*AKT2_INH)*. When this perturbation was applied, an additional invasive stable state was reached when compared to the unperturbed setup (Fig. 2E), therefore leading to a total of three pro-invasive and two pro-apoptotic stable states. On average, Boolean states of phenotype nodes favored invasiveness, but analysis with BooLEVARD showed that the two pro-apoptotic stable states exhibited notably more signaling intensity than their three pro-invasive counterparts (Fig. 2P). These results are further supported by experimental evidence, which demonstrates that AKT2 inhibition has a negative impact on invasion and colony formation in colorectal cancer, whereas AKT1 inhibition has no effects^32^. Consistently, BooLEVARD also showed that the inhibition of AKT1 resulted in little to no effect on invasion. Interestingly, BooLEVARD predicted *AKT1_ACT* to have a stronger pro-invasive effect than *AKT2_ACT* (the anti-apoptotic effects of both perturbations remain similar), although stable states analysis resulted in overlapping effects. AKT proteins regulate key cellular processes, including protein synthesis, proliferation, invasion, and inflammation^33^. In cancer, AKT1 overactivation has been linked to tumor growth during earlier invasion stages, whereas AKT2 has been shown to be more involved in facilitating distant metastases^34^.

**Fig. 3:**
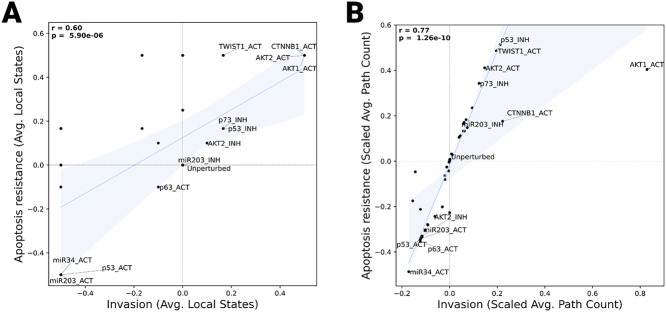
Correlated dynamics between invasion and apoptosis phenotypes. **A** A scatter plot depicting the impact of additive perturbations on the Boolean states of the *Invasion* (*X*-axis) and Apoptosis (*Y*-axis) phenotype nodes. **B** A scatter plot depicting the impact of additive perturbations on the path counts triggering the activation or inhibition of the *Invasion* (*X*-axis) and *Apoptosis* (*Y*-axis) phenotype nodes. The points represent *Invasion* and *Apoptosis* scores for each perturbation (*n* = 48) and the unperturbed setup, which are calculated as the average of *Invasion* and 1-*Apoptosis* local states (**A**) and path counts (**B**), min-max scaled (with reference at the unperturbed setup). Pearson correlation coefficient (*r*) and associated *p* values are annotated in the upper-left corner of each plot. The regression line of best fit and the estimate of the 95% confidence interval are displayed in blue.

Finally, to investigate how the invasion and apoptosis variables relate to one another using both methods, we investigated the score distributions obtained from the stable states and BooLEVARD analyses, respectively (Fig. 3, Supplementary Fig. 6). Although the stable state analysis already showed a correlation between invasion and apoptosis (represented as apoptosis resistance in the figure), stronger Pearson correlation was displayed by BooLEVARD’s results (*r* = 0.60, *p* = 5.90e-6 vs. 0.77, *p* = 1.26-e10). This further emphasized the increased granularity of BooLEVARD’s output and its value in producing results that contribute to improved interpretation of biological observations.

### BooLEVARD scales efficiently with increasing model complexity

To evaluate BooLEVARD’s scalability and computational efficiency across a wide spectrum of network configurations, we constructed randomized Boolean models of varying sizes, specifically *N* = 20, 40, 60, 80, and 100 nodes (Supplementary Code 3). Within each size category, we stratified networks by connectivity, applying a discrete uniform distribution of incoming edges from 1 up to a maximum in-degre (*k*_max_) of 2, 3, 4, and 5. Each group included 20 Boolean models. All networks were seeded with three designated input nodes to ensure consistency. For each model, BooLEVARD was tasked with exhaustively enumerating every path that led to the activation of each node in all reachable stable states.

**Fig. 4:**
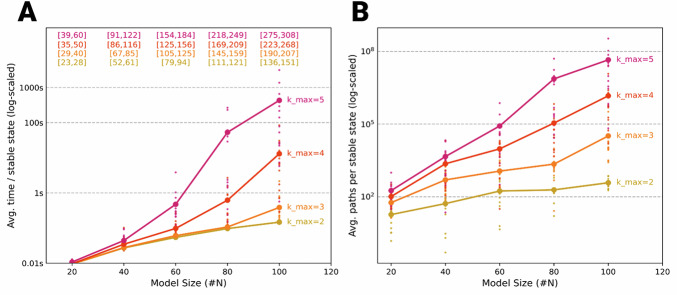
BooLEVARD’s performance and scalability. **A** Log-scaled marker-line chart of BooLEVARD’s average simulation time per stable state for path enumeration in random Boolean networks of size *N* = 20, 40, 60, 80, and 100 nodes with a discrete uniform in-degree distribution from 1 to 2 (pink), 3 (red), 4 (orange), and 5 (yellow). Individual models are overlaid as smaller scatter points. Above each data point, the [min, max] range of edges for the network configuration is annotated in the corresponding color group. *Y*-axis labels correspond to log-untransformed time. **B** Log-scaled marker-line chart of BooLEVARD’s average path enumeration per stable state in random Boolean networks of size *N* = 20, 40, 60, 80, and 100 nodes with a discrete uniform in-degree distribution from 1 to *k*_max_ = 2 (pink), 3 (red), 4 (orange), and 5 (yellow). Individual models are overlaid as smaller scatter points. *Y*-axis labels correspond to log-untransformed path counts.

The resulting performance profiles (Fig. 4A, Supplementary Data 1) revealed a clear dependence of simulation time on both network size and in-degree. In sparsely connected networks (*k*_max_ = 2 or 3), BooLEVARD completed path enumeration per stable state in under 1 s on average, even while enumerating between 10^3^ or 10^4^ distinct paths per state (Fig. 4B). As network size grew to *N* = 90 and 100 with higher connectivity (*k*_max_ = 4 or 5), runtimes extended from several seconds up to 1 h in the most extreme cases. This corresponded to a surge in path counts on the order of 10^6^ or 10^8^ per steady state. Despite this steep increase, BooLEVARD maintained robust performance, efficiently handling larger networks with substantial connectivity and delivering exhaustive path enumeration within practical timeframes.

### BooLEVARD adds an extension to signal transduction analysis in Boolean models

Several existing methods enable the exploration of signal propagation and influence in Boolean models, and were compared to BooLEVARD (Table 1). For example, the Logic Hypergraph framework^18^ provides a powerful formalism to represent complex logical relationships by encoding multi-input regulatory rules as hyperedges. This allows for the classification of nodes based on their regulatory influence and the exhaustive exploration of signal flow. A related approach is the concept of ESMs^19^, which defines minimal, irreducible subgraphs capable of functionally transmitting a signal from a source to a target node. The Logic Backbone method^20^ takes a different perspective by identifying minimal sets of regulatory interactions that are structurally sufficient to determine the state of each node, producing a compact subgraph that captures the essential dependencies within the network. Pint^12^ is a tool designed for the static analysis of large-scale automata networks, enabling fast computation of reachability, identification of necessary transitions, and detection of cut sets that prevent signal propagation to specific targets.

**Table 1:** Comparison of methods for analyzing signal propagation in Boolean models

Feature	Explicit Path Count	State-specific	Signed Paths	Workflow	Ref.
BooLEVARD	Yes	Yes	Yes	cDNFs, Drivers, Driver Simplifications, Path Counting	-
Logic Hypergraph	No	No	No	Build Hyperedges, exhaustive DFS of hyperwalks	^18^
ESMs	No	No	No	Expand graph, ILP/DFS for minimal subgraphs	^19^
Logic Backbone	No	No	No	Expand gadgets, Stable motifs, Prune edges	^20^
Pint	Yes	No	Yes	Static analysis, Symbolic reachability reduction	^12^

Explicit Path Count indicates whether the method quantifies distinct paths; State-specific refers to the ability to perform the analysis condition on individual stable states; Signed Paths denotes whether activatory and inhibitory influences are distinguished; the Workflow column summarizes the main computational steps employed by each method.

However, the computational demands associated with path or signal propagation analysis often require users to impose constraints, such as limiting path length, restricting computation time, or relying on heuristic strategies. Path enumeration within a hypergraph suffers from combinatorial explosion, and ESM characterization using ILP or DFS becomes intractable in large networks. While Pint offers scalable reachability analysis, it may overapproximate the set of executable transitions by including paths that are not logically feasible under certain conditions. Logic Backbone, in turn, provides a minimal and interpretable structure, but abstracts away alternative or redundant paths and does not capture the number or cumulative influence of parallel signals.

BooLEVARD was developed to address these limitations by providing a unified framework for path-based signal analysis that is both quantitative and sign-aware. BooLEVARD explicitly enumerates logically valid activatory and inhibitory paths from any source to a given target directly from the Boolean rules by exhaustively counting signed paths across the stable states of a Boolean model. Moreover, through stable-state-specific driver formulation, subsequent driver simplification, and reuse of intermediate results from previously evaluated nodes within each stable state, BooLEVARD avoided redundant computations, enhancing its scalability in models of moderate sizes. This altogether emphasizes the distinctive focus of BooLEVARD on path-based logic and its ability to quantify the cumulative influence of activatory and inhibitory signals.

## Discussion

Boolean models, due to their simplicity and ability to represent cell signaling events, represent a powerful resource for studying the complex molecular dynamics of biological processes. However, their discrete nature limits their capacity to capture continuous aspects of cellular signaling, such as signal strength or the degree of activation or repression of biological entities^4^. While existing tools and methods like Pint, Logic Hypergraphs, ESMs, and Logic Backbone provide means to explore signal transduction, the computational demands of path exploration often force users to set limitations, such as maximum path length or computation time, to make calculations feasible^12,18–20^. As a result, a consistent workflow for assessing signal transduction strength in Boolean models has yet to be elucidated.

To fill this methodological gap, we introduced BooLEVARD, an efficient tool for computing the number of paths leading either to node activation or repression. BooLEVARD allows the redefinition of node Boolean states to represent the set of non-redundant paths directly influencing the observed Boolean outcome. Additionally, BooLEVARD supports the introduction of node perturbations within a framework compatible with path calculations. To ensure interoperability with other tools, BooLEVARD reads Boolean models in the standard BoolNet format^35^ and allows for the models to be exported back to the same format. Additionally, since the path collection computed by BooLEVARD is an intrinsic feature of Boolean models, the tool can be applied not only to analyze models representing biological processes but also to study signal strength in any Boolean model.

In the first use case, the usage of BooLEVARD in the metastatic model highlighted the importance and added value of focusing on signaling strength to better understand cell-fate decisions. Furthermore, the results were more precise and better aligned with biological findings, in comparison to stable state analysis, as supported by the existing literature. The semi-continuous nature of BooLEVARD’s output enabled a more accurate identification of the most impactful perturbations, providing insights into which possible drug target nodes are likely to be more effective and which node mutations may be more strongly associated with metastatic risk, thus complementing current approaches toward the use of computational models in personalized medicine efforts^36,37^. In the second use case, our scalability analysis demonstrated BooLEVARD’s ability to handle increasingly large and densely connected Boolean models without sacrificing exhaustiveness or accuracy. By systematically varying model size and maximum in-degree, we showed that BooLEVARD maintains subsecond runtimes for moderate connectivity and remains tractable even for networks of 100 nodes and higher maximum in-degrees, completing path enumeration in under a minute in most cases.

In the future, several directions could further enhance BooLEVARD’s capabilities and broaden its applicability. Incorporating feedback loop analysis would allow the tool to capture recurrent motifs and self-sustaining signaling circuits. Similarly, the ability to handle cyclic attractors without losing scalability could be explored using trap-space approximations to define oscillatory subspaces and then apply random path sampling within those spaces, trading exhaustive enumeration for performance. Another promising direction involves introducing path weighting schemes, where users could define weights based on biological assumptions, edge strength, or decay functions, enabling more nuanced quantification of signaling strength. Finally, while BooLEVARD already demonstrates efficient performance, larger-scale applications could benefit from optimization through compiled backends and parallel computing strategies. In conclusion, BooLEVARD introduces a novel, interpretable, and scalable approach to quantify signal transduction in Boolean models by tracing the structure and intensity of paths in Boolean models. Its ability to resolve subtle differences in signaling logic across stable states and perturbations enhances biological insight and offers a promising avenue for applying Boolean models in predictive and translational contexts. As it continues to evolve, BooLEVARD is well-positioned to become a powerful component of the Boolean modeling toolbox in systems biology and beyond.

## Methods

### Requirements

BooLEVARD is available in Python 3 as a PyPi package, providing an efficient and user-friendly interface that can be implemented in Jupyter Notebooks. Detailed installation instructions and a quickstart tutorial are available in the package’s GitHub repository (https://github.com/farinasm/boolevard).

### Inputs and the BooLEV class

BooLEVARD takes Boolean models in BoolNet format (*.bnet*) as input^35^, which are loaded and managed as objects of the BooLEV class. Each BooLEV object provides structured access to key model information, including a list of nodes, a dictionary storing logical rules in canonical Disjunctive

Normal Form (cDNF)^38^, a dictionary for the negated rules in cDNF (referred to as cNDNF for consistency), a pandas data frame containing the model’s stable states, and a data frame that integrates all this information. This structure enables efficient exploration and downstream analysis of Boolean models within BooLEVARD. The stable states of the model are automatically computed by the Most Permissive Boolean Network (mpbn) package (most-permissive update scheme)^11^, and the Python Boolean Networks (pyboolnet) package (synchronous and asynchronous update schemes)^35^, while the cDNFs and cNDNFs are determined with the Python Electronic Design Automation (PyEDA package)^39^.

**Fig. 5:**
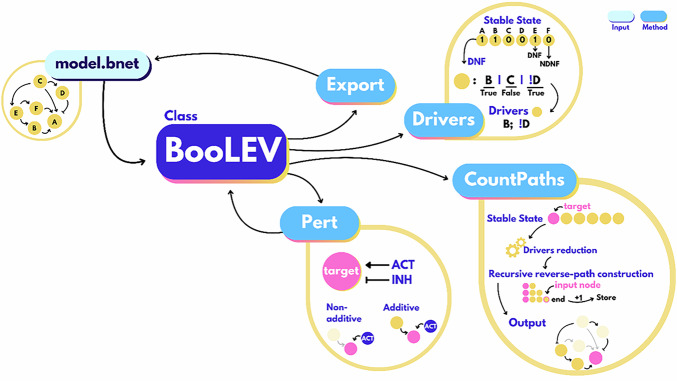
Overview of the BooLEVARD package. BooLEVARD processes Boolean models in *.bnet* format by converting them into *BooLEV* objects, integrating the *Export*, *CountPaths*, *Drivers*, and *Pert* methods. The *Export* method allows users to save *BooLEV* objects back as *.bnet* files, ensuring compatibility with other tools. The *CountPaths* method performs BooLEVARD’s core function – counting the number of activatory and inhibitory paths of a specific node within the stable states of the model. Stable-state-specific *drivers* (conjunctive blocks within a Boolean equation in cDNF) of each node are calculated using their cDNFs (for active nodes) or cNDNFs (for inactive nodes). *Drivers* can also be computed by calling the *Drivers* method. The *drivers* dictionary is simplified by applying iterative unit-lateral propagation, and paths are recursively reversed-constructed and stored in a counter, being this the output of the *CountPaths* method. The *Pert* method enables users to introduce perturbations into *BooLEV* objects by modifying the Boolean equations of target nodes. Perturbations can be either additive, where the perturbation is incorporated into the existing logical rule, or non-additive, where the original equation is completely substituted to exclusively reflect the perturbation’s effect. The updated *BooLEV* can be exported back to *.bnet* format.

BooLEV objects implement four methods: *CountPaths*, *Drivers*, *Pert*, and *Export*. The package overview of BooLEVARD is illustrated in Fig. 5.

### Recursive reverse-path construction and enumeration

**Fig. 6:**
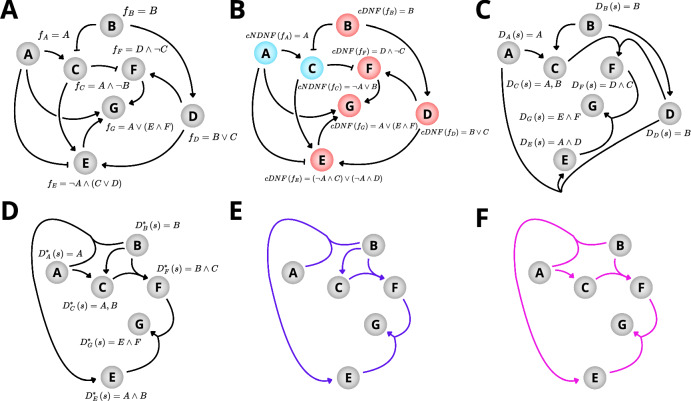
Schematic representation of a BooLEVARD’s path reconstruction. **A** Schematic of a Boolean model composed of seven nodes (A–G) and their Boolean equations. Arrows and T-arrows represent positive and negative interactions, respectively. **B** Visualization of a stable state. Nodes set to 1 (active) are highlighted in red and display their cDNFs, and nodes set to 0 (inactive) are highlighted in blue and display their cDNFs. **C**, **D** Representation of the drivers and simplified drivers set for the stable state, represented in (**B**). **E**, **F** Representation of the two complex paths allowing G activation in the stable state, represented in (**B**).

BooLEVARD’s main feature is to count the number of paths leading to the activation or inactivation of a specific node within a stable state reached by a Boolean model (both selected by the user), achieved using the CountPaths method. To determine the number of paths, BooLEVARD employs a recursive reversed-path construction strategy, building paths from the target back to the input nodes of the network (i.e., those with no incoming regulation). The first step in this process involves checking the Boolean state of each node in the chosen stable state to retrieve either the cDNFs for active nodes or the cNDNFs for inactive nodes (Fig. 6A, B). The cDNF provides a non-redundant, maximally simplified representation of a Boolean equation as a disjunction of conjunctions, where each conjunctive block represents a potential step in the transduction pathway leading to the node’s Boolean state^38^. These blocks serve as the fundamental components of the paths analyzed by BooLEVARD. The *CountPaths* method then uses the c(N)DNFs to compute a dictionary of drivers (Fig. 6C). For a given stable state, cDNFs and cNDNFs are scanned, and every conjunctive block that evaluates to true is selected. Then, the drivers for the state are extracted as the exact set of literals in each such true clause, because only their joint truth suffices to force the rule’s target Boolean state. This computation is independently available through the *Drivers* method of BooLEV objects. Once the drivers are computed, an iterative unit-lateral propagation, analogous to transitive reduction^40^, is applied (Fig. 6D). At each round, any driver that has collapsed to a single variable is substituted verbatim into all downstream drivers. This flattens the dependency structure—eliminating trivial intermediates—while preserving the original signal transduction logic and reducing the complexity of subsequent path enumeration. Following this, BooLEVARD uses the simplified drivers of the target node, each being the next upstream unit of a potential path (Fig. 5).

The resulting set of partial paths is analyzed to ensure that none of the paths are repeated; any duplicates are discarded. This process is applied recursively, counting until all paths are fully processed, that is, until they either form a loop or reach an input node, a condition that is checked at every elongation step (Fig. 6E, F). To increase efficiency, BooLEVARD reuses previously computed path information from upstream nodes within the same stable state, avoiding redundant calculations. Accordingly, the tool identifies acyclic paths, which are defined as simple or complex. Simple paths are those built entirely from single-literal driver blocks, whereas complex paths include at least one expansion from a driver of at least two literals, generating multiple sub-paths via a Cartesian product at that step. A graphical overview of the original and optimized drivers, along with the derived paths, is shown in Fig. 6.

### Model perturbations

**Fig. 7:**
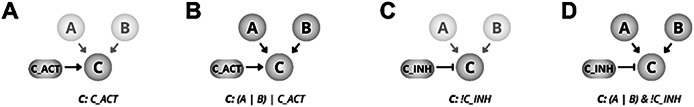
Schematic representation of the perturbation types implemented in BooLEVARD. **A** Schematic of a non-additive activation. **B** Schematic of an additive activation. **C** Schematic of a non-additive inhibition. **D** Schematic of an additive inhibition.

Tools for processing Boolean models often include functions for simulating perturbations, typically node knockouts (KOs) or ectopic expressions (EEs)^10,11,13^. These perturbations are usually implemented by replacing the target node’s Boolean equation with either 0 (for KOs) or 1 (for EEs). However, since BooLEVARD constructs signaling paths directly from the model’s Boolean equations, this replacement approach is not feasible. To address this, BooLEV objects include the *Pert* method, which extends the range of perturbations from two to four types: two forms of activation and two forms of inhibition, each categorized as either additive or non-additive. Upon non-additive perturbations, the target node’s Boolean equation is substituted by a Boolean formalism that reflects only the regulation triggered by the perturbation. Additive perturbations determine the state of the target node similarly, but the effects of the perturbation are incorporated into the target node’s original Boolean equation. To implement the perturbations, BooLEVARD creates a dedicated perturbation node that either positively (activations) or negatively (inhibitions) influences the target node. The Boolean equation of the target node is then updated to account for the user-defined additive or non-additive perturbation (Fig. 7). The resulting perturbed version of the Boolean model is stored as a BooLEV object.

Notably, BooLEVARD may also be applied to models lacking explicit input nodes by implementing perturbations, which serve as synthetic inputs via dedicated surrogate nodes. This formulation allows the user to represent external stimuli or environmental conditions and thereby assess their impact on the network’s internal logic. Furthermore, the same framework can be used to infer the number of paths connecting any two nodes of interest.

## Supplementary information

Supplementary material

Supplementary Data 1

Supplementary Code 1

Supplementary Code 2

Supplementary Code 3

## Data Availability

BooLEVARD is publicly available as a PyPi package at https://github.com/farinasm/boolevard, with user documentation detailing installation procedures and a step-by-step usage guide. The package documentation, including the API reference, was generated using Sphinx^41^ to ensure clarity and ease of navigation, available at https://farinasm.github.io/boolevard/. To facilitate user onboarding, we provide several tutorials as Jupyter Notebooks, allowing users to explore BooLEVARD’s functionalities through a practical example. The source code used to generate the figures and supplementary figures presented in this paper is available at https://github.com/farinasm/boolevard/tree/main/paper.
