# Supplementary material for: Path-based quantification of activation and repression in Boolean models using BooLEVARD
Source: NPJ Syst Biol Appl. 2025 Nov 19;11:129. doi: 10.1038/s41540-025-00605-y (PMC12630683; doi:10.1038/s41540-025-00605-y)
Supplement: Supplementary file 1 — Supplementary material [file 41540_2025_605_MOESM1_ESM.pdf]

Supplementary Material

BooLEVARD: Boolean Logical Evaluation of Activation and Repression in Directed pathways

- BooLEVARD's GitHub repository: https://github.com/farinasm/bolevard
- Documentation and API reference: https://farinasm.github.io/boolevard/
- Supplementary material: https://github.com/farinasm/boolevard/tree/main/paper

BooLEVARD is publicly available as a PyPi package at, with user documentation detailing installation procedures and a step-by-step usage guide. The package documentation.

Supplementary Code 1. Jupyter notebook containing the source code for generating the figures presented in the first use case of the study.

Supplementary Code 2. Jupyter notebook containing a script with a generic analysis of Boolean models available at the Cell Collective repository.

Supplementary Code 3. Jupyter notebook containing the source code for generating the figures presented in the second use case of the study.

Supplementary Data 1. CSV document containing the simulation times for the analysis assessed in the second use case of the study.

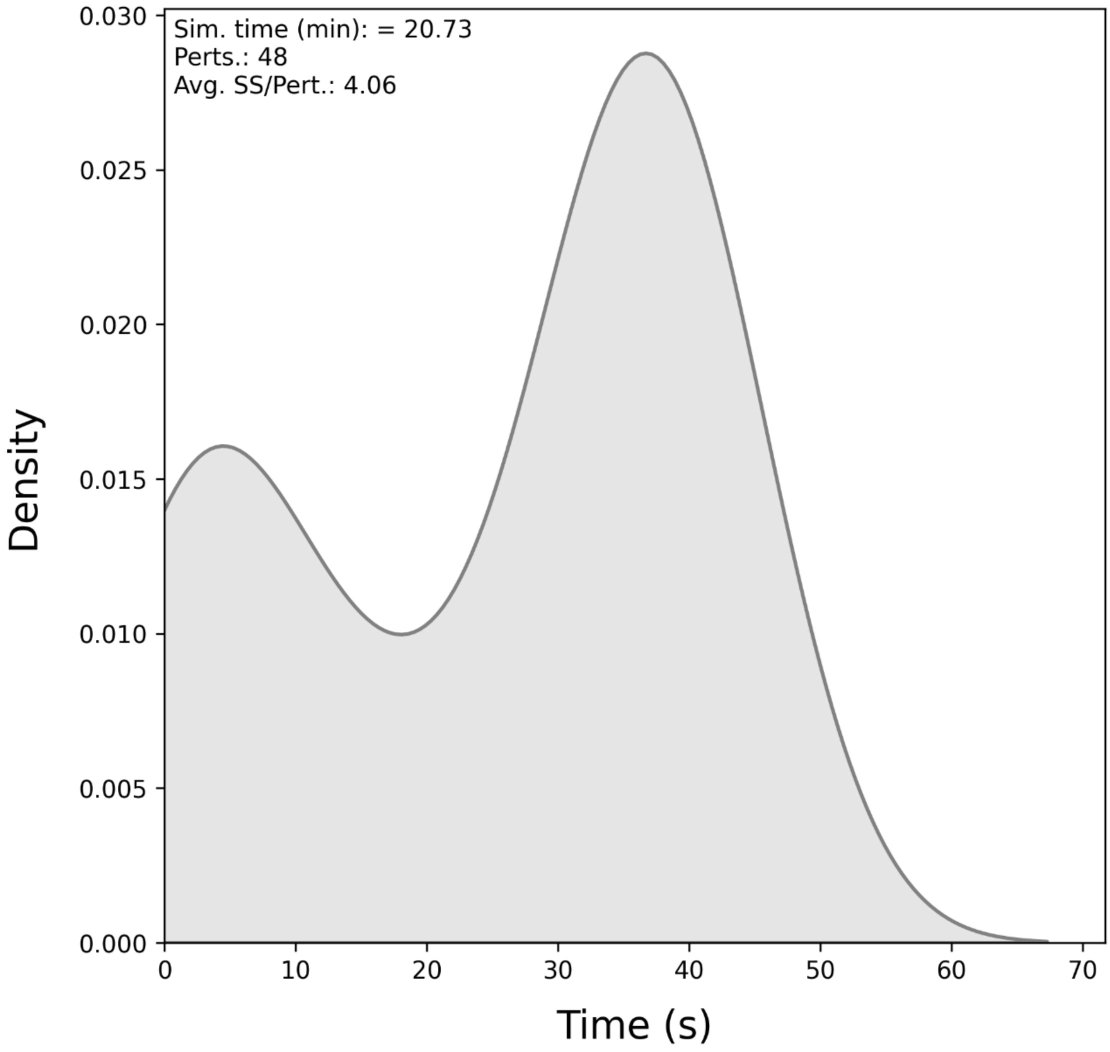

Supplementary Figure 1. Density plot illustrating the distribution of BooLEVARD's simulation times for each processed stable state.

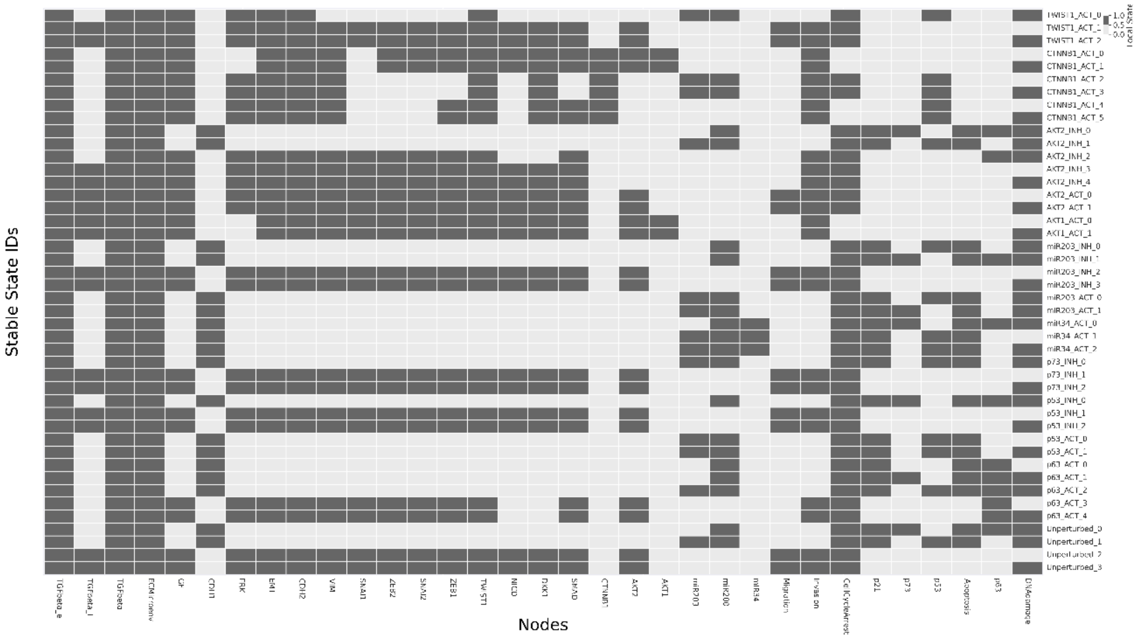

Supplementary Figure 2. Heatmap showing the stable states reached by the model upon perturbations discussed in Figure 4E-P. The local states of the input nodes driving each stable state are displayed in dark gray (ON) or light gray (OFF). ECMicroenv input node was set to 1 as a constraint in every perturbation.

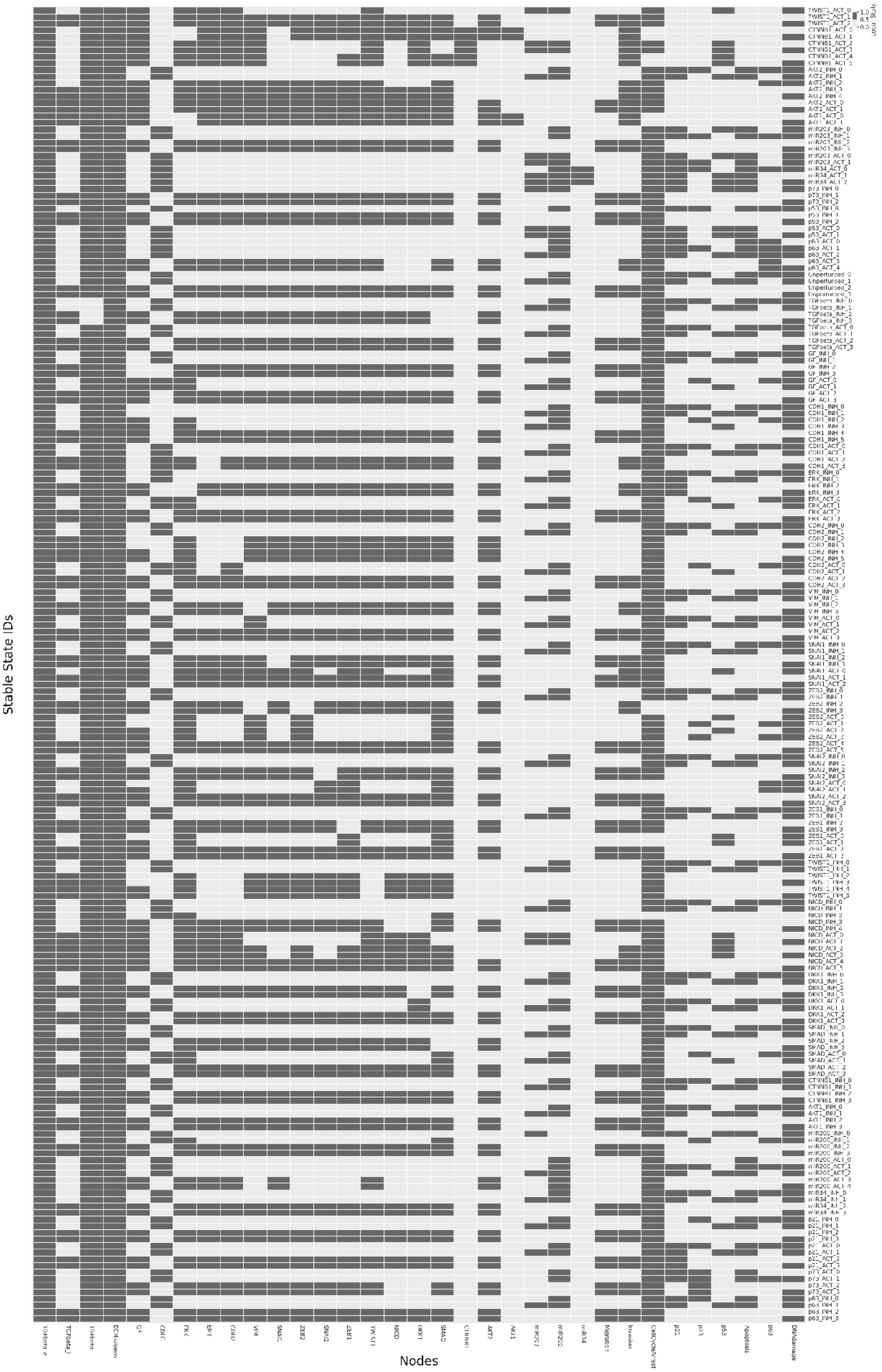

Supplementary Figure 3. Heatmap showing the stable states reached by the model upon every performed perturbation. The local states of the input nodes driving each stable state are displayed in dark gray (ON) or light gray (OFF). ECMicroenv input node was set to 1 as a constraint in every perturbation.

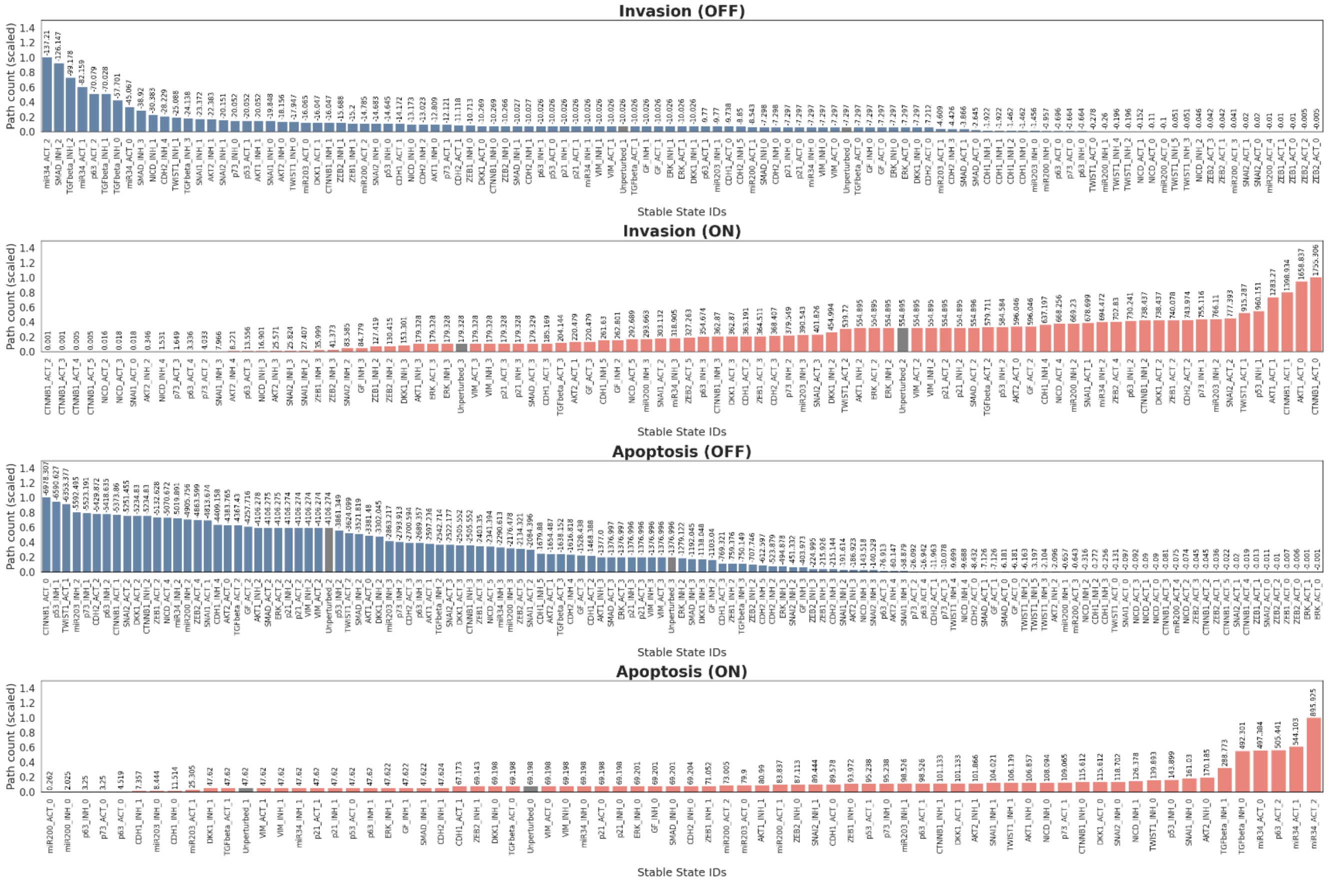

Supplementary Figure 4. Barplots illustrating the min-max scaled path counts for the Invasion and Apoptosis across each stable state reached upon the perturbations performed. From top to bottom: path counts leading to the inactivation of Invasion, path counts leading to the activation of Invasion, path counts leading to the inactivation of Apoptosis, path counts leading to the activation of Apoptosis. Red and blue bars represent stable states reached upon the perturbations resulting in an active or inactive Boolean state of the phenotype node. Gray bars represent path counts of stable states reached upon the unperturbed set up. Non-scaled path counts triggering activation (positive) or inactivation (negative) are annotated on top of the bar of each perturbation.

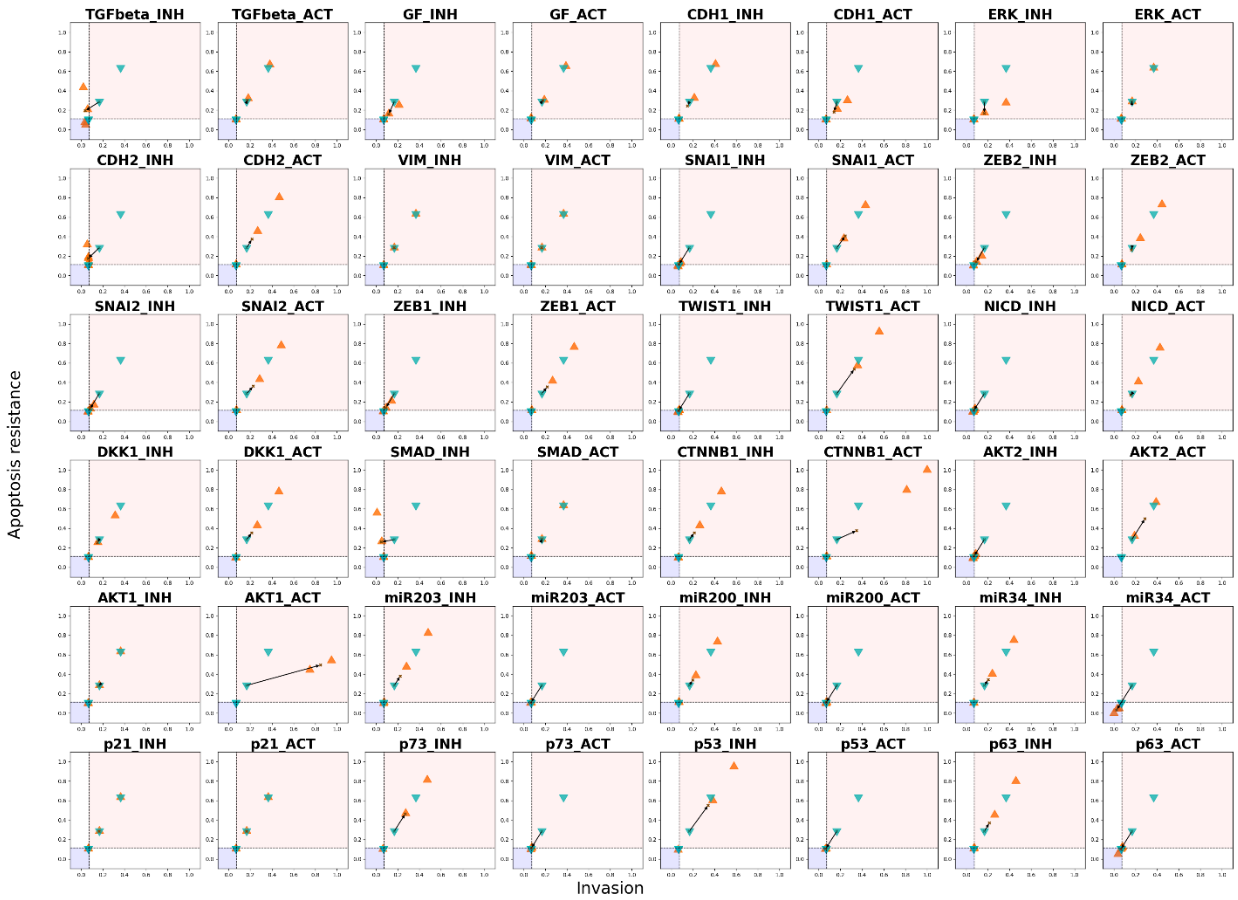

Supplementary Figure 5. Single-perturbation-induced invasiveness fate transitions represented by the scaled path counts upon every perturbation performed. Each plot corresponds to a specific perturbation. The x-axis represents stable-state-wise min-max scaled path counts for the Invasion node, and the y-axis shows apoptosis resistance as 1 - the stable-state-wise min-max scaled path counts for the Apoptosis node. Blue and brown triangles indicate the stable states of the unperturbed and perturbed models, respectively. Since the model has one input node allowed to vary between 1 or 0, and two stable states in either condition, there are four blue triangles (two superimposed and not visible in the figure). Blue and brown crosses represent the averaged stable states path counts for the unperturbed and perturbed models, respectively. Black arrows illustrate the transitions in invasive fate from the unperturbed to the perturbed setups. Dashed horizontal and vertical lines separate sectors with negative and positive path counts (i.e. inactive and active states) for the Invasion and Apoptosis nodes, respectively. Red and blue areas display pro-invasive (i.e. Invasion = 1, Apoptosis = 0) and pro-apoptotic (i.e. Invasion = 0, Apoptosis = 1).

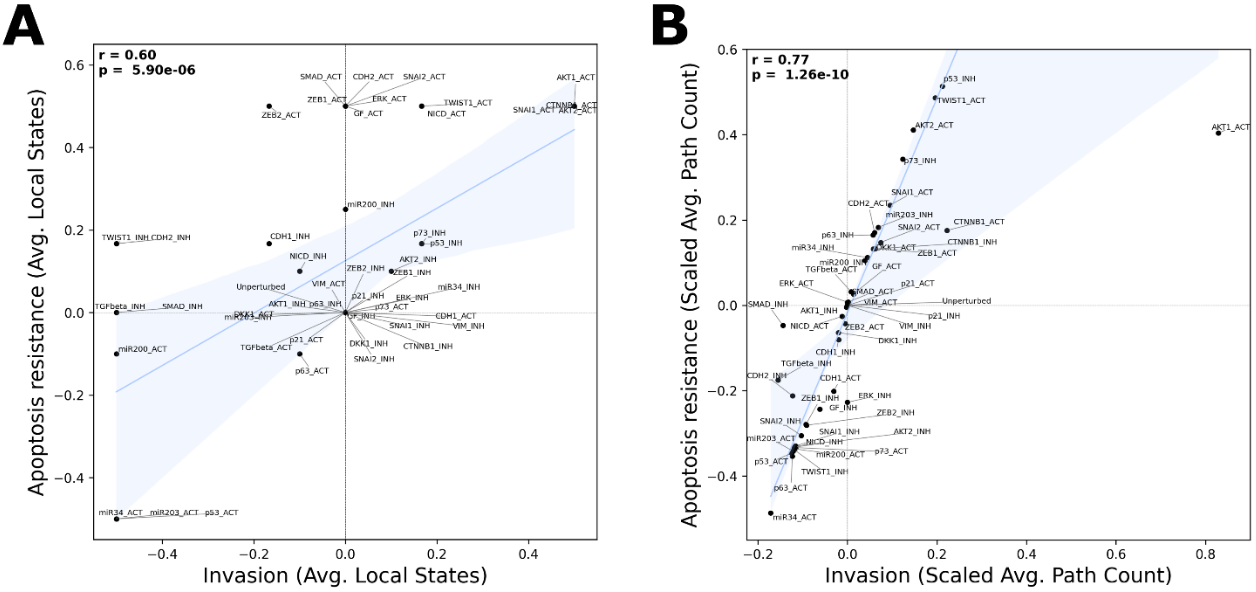

Supplementary Figure 6. A. A scatter plot depicting the impact of additive perturbations on the Boolean states of the Invasion (X-axis) and Apoptosis (Y-axis) phenotype nodes. **B.** A scatter plot depicting the impact of additive perturbations on the path counts triggering the activation or inhibition of the Invasion (X-axis) and Apoptosis (Y-axis) phenotype nodes. The points represent Invasion and Apoptosis scores for each perturbation (n = 48) and the unperturbed setup, which are calculated as the average of Invasion and 1-Apoptosis local states (A) and path counts (B), min-max scaled (with reference at the unperturbed set up). Pearson correlation coefficient (r) and associated p-values are annotated in the upper-left corner of each plot. The regression line of best fit and estimate of 95% confidence interval are displayed in blue.
